# DAILY AFFECT AND SLEEP AMONG FAMILY CARE PARTNERS OF PEOPLE LIVING WITH DEMENTIA

**DOI:** 10.1093/geroni/igae098.3374

**Published:** 2024-12-31

**Authors:** Yeonsu Song, Kevin Kuehn, Nicole Lewis, Alexander Erickson, Brent Mausbach, Jennifer Martin, Raeanne Moore

**Affiliations:** University of California Los Angeles, Los Angeles, California, United States; University of California San Diego, La Jolla, California, United States; University of California Los Angeles, Los Angeles, California, United States; VA Greater Los Angeles Healthcare System, Los Angeles, California, United States; University of California San Diego, La Jolla, California, United States; VA Greater Los Angeles Healthcare System, Los Angeles, California, United States; University of California San Diego, La Jolla, California, United States

## Abstract

Caregiving can be a positive and negative experience simultaneously. However, many of the family care partners of people living with dementia (PLWD) often experience poor sleep, leading to heightened levels of depression and caregiving strain. This study examined daily variations in positive and negative affect and the bidirectional role of sleep and daily affective states. A total of 35 care partners (mean age 63 years, 39% non-White, 81% women) completed ecological momentary assessment surveys of their positive and negative affect and subjective sleep three times a day for seven days. Variables included positive (currently feeling energetic, happy, motivated, rested, relaxed) and negative affect (currently feeling anxious, depressed, helpless, hopeless, nervous, tense, worried, worthless; range 1-5), and ‘good sleep’ (i.e., no trouble with falling asleep and staying asleep) on previous nights. Descriptive and mixed-effect logistic regressions were performed. Mean levels of positive affect were significantly lower in the evening compared to the morning/afternoon (F (df= 1, 491) = 54.24, p< 0.001); no time-of-day differences were found for negative affect (F (df= 1,492) = 1.33, p=0.25). Higher levels of mean positive affect were associated with a higher probability of good sleep (OR=2.23, 95%CI=1.26-3.94, p< 0.01). No significant relationships were found between negative affect and good sleep. This study demonstrates a within-day variation in positive affect among family care partners, which is related to the quality of sleep the night before. Understanding these dynamics is crucial for developing targeted interventions to improve the sleep and emotional well-being of care partners of PLWD.

